# P-35. Long-Term Healthcare Utilization and Expenditures for Infants with Invasive Bacterial Infections During Birth Hospitalizations

**DOI:** 10.1093/ofid/ofaf695.264

**Published:** 2026-01-11

**Authors:** Kristen Noble, Katharina Schley, Lisa Abramovitz, Sarah J Willis, Kyla hayford, Jennifer Moisi, Derek Weycker

**Affiliations:** Pfizer Pharma GmbH, Berlin, Berlin, Germany; Pfizer Pharma GmbH, Berlin, Berlin, Germany; Avalere Health, Boston, Massachusetts; Pfizer, Wakefield, Massachusetts; Pfizer, Wakefield, Massachusetts; Pfizer Vaccines, Collegeville, Pennsylvania; Avalere Health, Boston, Massachusetts; Indiana University School of Medicine, Indianapolis, Indiana

## Abstract

**Background:**

Invasive bacterial infections among newborns are associated with significant morbidity, mortality, and economic costs. While most newborns fully recover following the acute phase of illness, some develop long-term complications that require additional medical care. The objective of this real-world study was to estimate long-term healthcare utilization and expenditures among infants with bacterial meningitis or sepsis during their birth hospitalizations.

**Figure:**
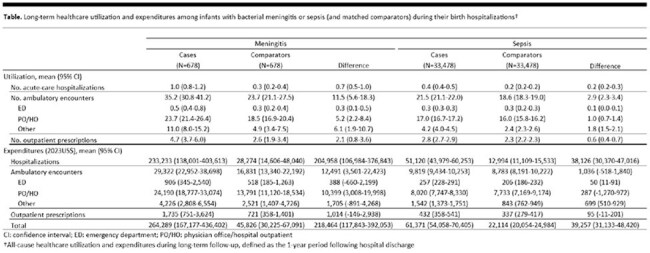

**Methods:**

A retrospective cohort design and the PharMetrics Plus Healthcare Claims Database (01/01/16-04/30/24) were employed. Study population comprised infants who, during their birth hospitalizations, had diagnosis codes for meningitis or sepsis due to bacterial pathogens as well as comparison infants without evidence of these conditions matched (1:1) on birth gestational age, birth weight, sex, high-risk conditions, and an estimated propensity score. Infants with evidence of meningitis and sepsis were included in the former subgroup. Study measures—including numbers of, and expenditures (2023 USD) for, all-cause hospitalizations, ambulatory encounters, and outpatient prescriptions—were ascertained during the 1-year period following discharge from the birth hospitalization and were annualized to adjust for differential follow-up.

**Results:**

Among infants with bacterial meningitis and matched comparators (N [pairs] = 678), 61% were born prematurely, 27% had birthweight < 1500 grams, and 56% had ≥1 high-risk condition; among those with sepsis (N [pairs] = 33,478), corresponding values were 48%, 20%, and 33%. Mean levels of healthcare utilization and expenditures during the 1-year follow-up period were markedly higher among infants with meningitis (total expenditures: by $218,464) or sepsis (total expenditures: by $39,257) versus matched comparison infants; differences were largely attributable to acute-care hospitalizations (Table).

**Conclusion:**

Invasive bacterial infections among infants place a substantial burden on the US healthcare system, including treatment of acute illness as well as associated long-term complications. Interventions targeting the prevention of infant bacterial infections have the potential to yield significant cost offsets.

**Disclosures:**

Kristen Noble, MD, PhD, Pfizer: Advisor/Consultant Katharina Schley, Dr. rer pol., Pfizer: Employee|Pfizer: Stocks/Bonds (Private Company) Lisa Abramovitz, MPH, Pfizer Inc.: Advisor/Consultant Sarah J. Willis, PhD, MPH, Pfizer, Inc.: Stocks/Bonds (Public Company) Kyla hayford, PhD, Pfizer: Stocks/Bonds (Public Company) Jennifer Moisi, PhD, Pfizer Vaccines: Employer|Pfizer Vaccines: Stocks/Bonds (Public Company) Derek Weycker, Ph.D., Pfizer Inc.: Grant/Research Support

